# Clinical and uterine cervix characteristics of women with *Mycoplasma* and *Ureaplasma* in genital discharge

**DOI:** 10.1590/1806-9282.20240045

**Published:** 2024-07-19

**Authors:** Margarida Santos Matos, Maria Clara Andrade Teles da Silva, Milena Bastos Brito, Ana Katherine Gonçalves

**Affiliations:** 1 Bahiana School of Medicine and Public Health, Department of Women's Health – Salvador (BA), Brazil.; 2 Universidade Federal da Bahia, School of Medicine of Bahia, Department of Gynecology and Obstetrics – Salvador (BA), Brazil.; 3 Universidade Federal do Rio Grande do Norte, Postgraduate Program in Health Sciences – Natal (RN), Brazil.; 4 Universidade Federal do Rio Grande do Norte, Postgraduate Program in Applied Sciences to Women's Health – Natal (RN), Brazil.; 5 Universidade Federal do Rio Grande do Norte, Department of Gynecology and Obstetrics – Natal (RN), Brazil.

**Keywords:** Mycoplasma, Uterine cervicitis, HPV, Cervix

## Abstract

**OBJECTIVE:**

The objective of this study was to assess the clinical and uterine cervix characteristics of patients displaying vaginal discharge with positive results for *Mycoplasma* sp. and/or *Ureaplasma* spp.

**METHODS:**

An analytical cross-sectional study involving women aged 18–45 years was conducted. Microbiological assessments included *Ureaplasma* and *Mycoplasma* cultures, as well as human papillomavirus hybrid capture using ecto and endocervix swabs. All tests were two-tailed, and significance was set at p<0.05.

**RESULTS:**

Among 324 women, *Ureaplasma* prevalence was 17.9%, and *Mycoplasma* prevalence was 3.1%. The *Ureaplasma*-positive group exhibited a higher frequency of urinary tract infections (39.1 vs. 19%, p=0.002) and human papillomavirus (39.7 vs. 12.8%, p≤0.001) compared with controls. The *Mycoplasma*-positive group showed a higher frequency of non-contraceptive use compared with controls (66.2 vs. 30.0%, p=0.036). Abnormal colposcopic findings were more prevalent in the *Mycoplasma/Ureaplasma*-positive group than in controls (positive: 65% vs. control: 35%, p=0.001). Pap smear findings did not differ between the groups.

**CONCLUSION:**

*Ureaplasma* spp. was associated with urinary tract infections and human papillomavirus, while the presence of *Mycoplasma* sp. was linked to reduced contraceptive use. When analyzing both pathogens together, a higher frequency of abnormal colposcopic findings was observed, with no difference in cytological findings in the positive group.

## INTRODUCTION

The prevalence of vaginal colonization by *Mycoplasma* sp. and *Ureaplasma* spp. among women tends to increase post-puberty, correlating with the number of sexual partners over their lifetime. While some authors characterize these microorganisms as commensal residents, they are also linked to various pathological conditions, including premature birth, vaginal discharge, urethritis, pelvic inflammatory disease, and infertility^1-3^.

Exposure of the cervicovaginal epithelium to *Mycoplasma* sp. and *Ureaplasma* spp. may give rise to a persistent intracellular infection, potentially leading to tissue damage mediated by inflammatory cytokines. Although the relationship between human papillomavirus (HPV) and these microorganisms is not conclusively established, the nature of the infection they cause allows for both direct interaction with HPV during co-infection of a single cell and indirect interaction through cytokine responses^4^.

Several studies indicate that the presence of *Mycoplasma* bacteria heightens the risk of more severe cervical lesions, such as low- and high-grade intraepithelial lesions^5^. In addition, women with abnormal cervical cytologies exhibit a 17.6 times greater risk for co-infection with *Mycoplasma hominis* and *Ureaplasma urealyticum* ^6^.

However, studies assessing the relationship between *Ureaplasma*/*Mycoplasma* and cervical cell changes are controversial. In 2018, a study examined the association between *M. hominis* infection and abnormal cervical cells but found no correlation between bacterial infections and abnormal cervical cytology^7^. Another study investigated the relationship between *Mycoplasma*, *Ureaplasma*, and HPV infections in sex workers, also failing to identify a correlation between *M. hominis*, *U. urealyticum*, and HPV infection^8^.

Contrary to these findings, it has been observed that high-risk HPV (hr-HPV) infection is a necessary cause of cervical cancer. However, other common microbes in the lower genital tract may enhance hr-HPV infection and cervical cytopathy^9^. The association of co-infection between HPV and sexually transmitted infections was compared using cervical samples from women with cervical dysplasia. Significant correlations were found between HPV, sexually transmitted infections, abnormal cervical cytology, HPV status, types of sexually transmitted infections, and the presence of *Ureaplasma* spp. and *M. hominis* ^10^.

To clarify the importance of *Mycoplasma*/*Ureaplasma* infection in the uterine cervix, this study aimed to describe the gynecological clinical data and uterine cervix alterations in patients presenting with vaginal discharge and positive results for *Mycoplasma* sp. and *Ureaplasma* spp.

## METHODS

An analytical cross-sectional study was conducted following the guidelines outlined in the STROBE statement^11^. The study took place between 2022 and 2025 in a private health service located in a region of northeastern Brazil with a Human Development Index (HDI) of 0.63.

The inclusion criteria encompassed women aged 18–45 years, with an active sexual life and complaints of non-physiological vaginal discharge. Exclusion criteria comprised menopausal status, genital bleeding during examination, immunosuppression, pregnancy, incomplete medical records, and hysterectomy.

Clinical data were collected by reviewing patient medical records and documented on a study-specific form. Variables included age, menarche age, number of sexual partners, obstetric history, parity, abortions, contraceptive method, urinary tract infections (UTIs), HPV status, characteristics of vaginal discharge (odor and itching), and cultures for *Ureaplasma* spp. and *Mycoplasma* sp. Reports related to colposcopic, cytological, and microbiological examinations were also consulted.

Colposcopic findings were categorized as normal, abnormal, or miscellaneous. Cytological findings were classified as unsatisfactory, normal (including normal smears and inflammation/cytolysis), and abnormal (ASC-US, ASC-H, ACG, LIEBG, and LIEAG).

The sample selection involved individuals classified as "Positive for *Ureaplasma*" and/or "Positive for *Mycoplasma*" constituting the case group, and those labeled "Negative for *Ureaplasma*" and "Negative for *Mycoplasma*" forming the control group. In addition, the presence of *Candida* sp., *Gardnerella*, and HPV was also investigated.

Diagnosis of *Ureaplasma* and *Mycoplasma* was obtained through microbiological culture using semi-liquid medium A/3 and A/7 specific to these microorganisms. The Sabouraud-Agar culture was utilized for diagnosing fungi and hybrid capture for HPV. The presence of *Gardnerella* was indicated by the identification of clue cells in the Pap smear and the presence of an odor. Patients with dysuria or hematuria underwent a urine culture and antibiogram to evaluate urinary infection.

Numerical and categorical data from the collected information were tabulated and statistically analyzed using the SPSS (Statistical Package for the Social Sciences) program version 14.0 (SPSS Inc., Chicago, IL, USA). An inductive/inferential analysis was conducted to describe the population and compare the groups. The Student's t-test was employed for quantitative variables with a normal distribution, the Mann-Whitney test was used for non-normally distributed quantitative variables, and the chi-square test was used for variables with n>5, with Fisher's exact test applied when n<5 for qualitative variables. All tests were two-tailed, with a significance level set at 5% (p<0.05) and a confidence interval of 95%.

### Ethics

The study adhered to the ethical and legal standards outlined in Resolution 466/12 of the National Health Council and received approval from the research ethics committee of the Fundação Bahiana para Desenvolvimento das Ciências, under CAAE number 6333520.5.0000.5544. Furthermore, the study was conducted by the Declaration of Helsinki and its subsequent revisions.

## RESULTS

Initially, 404 patients were enrolled, with 80 subsequently excluded based on pre-established exclusion criteria. Ultimately, 324 women of reproductive age were selected, among whom 58 tested positive for *Ureaplasma* spp., 10 tested positive for *Mycoplasma* sp., and 256 had negative cultures for these microorganisms.

The prevalence rates were 3.1% (10/324) for *Mycoplasma* sp. and 17.9% (58/324) for *Ureaplasma* spp. Coital activity was more common among individuals aged 10–20 years (81.01%). Most reported having one to five sexual partners, with 67.1% having never been pregnant and 81.8% having no history of abortion. The majority (65.1%) used some form of contraceptive method. Notably, white discharge without odor or itching was prevalent in the sample (56.8%).

In the bivariate analysis, only the presence of HPV was associated with *Ureaplasma* infection, even after adjusting for confounding variables (OR: 17.42, 95%CI: 3.08–161.2, p=0.004). Regarding *Mycoplasma* infection, only the use of contraceptives proved to be a protective factor (OR: 0.23, 95%CI: 0.005–0.86, p=0.038). Among the patients studied, 211 (65.1%) were using a contraceptive method. The most used method was hormonal contraceptives (56.4%), in both its oral and injectable versions, followed by the variable of patients not using contraceptive methods (34.9%). The male condom was used for 52 (24.6%) of the patients' partners.

Analysis of co-infections revealed that the presence of *Ureaplasma* spp. occurred simultaneously with HPV infection in 39.7% (n=23) of cases, showing a significant difference (p=0.001) and a moderate strength of association (contingency coefficient=0.261). However, there is a higher likelihood of patients with non-physiological genital flow being negative for both infections, accounting for 87.2% (n=232) in our sample. Further examination of HPV positivity within groups revealed an uneven distribution (p=0.001), with a higher frequency of oncogenic HPV. In the *Ureaplasma*-positive group, the frequency of oncogenic HPV was 22.4% (n=13). Other infections did not show differences (Table 1).

**Table 1:** Description of data on the presence and absence of infections in the group of patients positive or negative for *Ureaplasma* sp.

Variables		n	*Ureaplasma* sp. n (%)		p-value	OR
			Negative	Positive		
HPV					0.001	
	Negative	267	87.2% (232)	60.3% (35)		1.44
	Positive	57	12.8% (34)	39.7% (23)		0.32
HPV					0.001	
	Non-oncogenic	11	3% (8)	5.2% (3)		
	Oncogenic	30	6.4% (17)	22.4% (13)		
	Non-oncogenic and Oncogenic	16	3.4% (9)	12.1% (7)		
Fungus					0.847	
	Negative	293	90% (240)	91.4% (53)		0.99
	Positive	30	9.4% (25)	8.6% (5)		1.0
Gardnerella					0.573	
	Positive	16	4.9% (13)	5.2% (3)		0.94
	Negative	308	93.6% (300)	2.4% (8)		1.0

Regarding co-infections involving HPV and *Mycoplasma*, it was noted that most cases tested negative for both HPV and *Mycoplasma*, comprising 83.1% (n=261) of the total cases. Among those cases that tested positive for *Mycoplasma*, 60% (n=6) were negative for HPV (OR=1.38). However, this association demonstrated a weak correlation (contingency coefficient=0.104) and lacked statistical significance between the groups (p=0.079). In the case of Fungi and *Gardnerella*, both exhibited higher percentages of negative cases in both groups, those with and without *Mycoplasma*. The analysis of the two infections revealed a greater occurrence of patients in the sample but did not indicate simultaneous infection with *Mycoplasma* (OR Fungi=1.14; OR *Gardnerella*=1.0). Nevertheless, no differences were identified in the studied groups for both fungi (p=0.235) and *Gardnerella* (p=0.403) (Table 2).

**Table 2:** Description of data on the presence and absence of infections in the group of patients positive or negative for *Mycoplasma* sp.

Variables		n	*Mycoplasma* sp. n (%)		p-value	OR
			Negative	Positive		
HPV					0.079	
	Negative	267	83.1% (261)	60% (6)		1.38
	Positive	57	16.9% (53)	40% (4)		0.42
HPV					0.001	
	Non-oncogenic	11	2.5% (8)	30% (3)		
	Oncogenic	30	9.2% (29)	10% (1)		
	Non-oncogenic and oncogenic	16	5.1% (16)	0% (0)		
Fungus					0.235	
	Negative	293	91.1% (285)	80% (8)		1.14
	Positive	30	8.9% (28)	20% (2)		0.45
Gardnerella					0.403	
	Negative	307	95.2% (298)	90% (9)		1.0
	Positive	16	4.8% (15)	10% (1)		0.48

## DISCUSSION

We observed a prevalence of 3.08% for *Mycoplasma* sp. and 17.9% for *Ureaplasma* spp. among women reporting non-physiological vaginal discharge. The higher prevalence of *Ureaplasma* compared with *Mycoplasma* aligns with previous studies where *Ureaplasma* spp. values ranged from 4.8 to 48.07%, while *Mycoplasma* sp. values varied between 0.8 and 23.4%^12-15^.

Contrary to this pattern, some studies reported a higher prevalence of *Mycoplasma* than *Ureaplasma*. For instance, Cardillo found 35.89% for *Mycoplasma* spp. and 25.54% for *U. urealyticum* ^15^, and Christofolini et al.^16^ found 11.3% for *M. hominis* and 0.94% for *U. urealyticum*. Such discrepancies in frequency among studies may result from variations in the populations studied and the techniques used to detect microorganisms^12,17^.

Furthermore, we identified an association between *Ureaplasma* infection and UTIs, consistent with a 2020 meta-analysis by Moridi et al.^17^ evaluating the prevalence of *M. hominis*, *Mycoplasma genitalium*, and *U. urealyticum* among Iranian couples. They reported a *U. urealyticum* prevalence of 17.53% and an *M. hominis* prevalence of 9.68%, noting higher infection rates in women with symptoms of genito-UTI compared with men with UTI (7.67% vs. 5.88 and 21.04% vs. 12.13%, respectively).

Recent studies propose a potential interference of *M. hominis*, *U. urealyticum*, and *Ureaplasma parvum* with HPV infection, leading to virus persistence. Some studies found a positive relationship between *U. urealyticum* and HPV, while others reported an overall correlation between *Ureaplasma* spp. and *M. hominis* with HPV9^10,12,14,18^. Our study aligns with these findings, showing a significant relationship between *Ureaplasma* spp. and the presence of HPV. However, contradicting these results, a 2018 Indonesian study concluded no connection between *Ureaplasma* and *Mycoplasma* sp. and HPV^8^.

Additionally, a study by Zdrodowska-Stefanow et al.^14^ demonstrated that the risk of HPV infection doubled when a woman was infected with any of the four species of *Mycoplasma*. In cases of concomitant *U. urealyticum* infection, the risk of HPV infection was 4.7 times higher. In contrast, another study from 2018 concluded that *Ureaplasma* spp. and *Mycoplasma* sp. were not linked to HPV^12^. The complexity of these relationships underscores the need for further research and genotyping of *Ureaplasma* spp. species^14^.

Regarding colposcopic findings, we noted a higher prevalence of abnormal results in positive patients, contrasting with a study reporting inconclusive colposcopy outcomes in patients with *U. urealyticum* and *M. hominis* ^15^. However, concerning cytological findings in our study, no significant association was observed. This aligns with the study by Effiana et al., which found no relationship between *M. hominis* and altered Pap smear results^7^. Yet, earlier studies demonstrated that *U. urealyticum*, *U. parvum*, and *M. hominis* may increase the risk of cytological changes in the uterine cervix^8,9,10^.

The influence of the vaginal microbiome on the development of neoplastic lesions in the uterine cervix has been documented in previous studies. While some reported the relevance of *Mycoplasma* sp. and *Ureaplasma* spp. in the context of cervical cancer^8,14,16^, others did not find a clear relationship with the onset and progression of CIN^8,17^. The varied findings emphasize the intricacies of these interactions and the need for future investigation in this field.

## CONCLUSION

*Ureaplasma* spp. was more prevalent and associated with UTI and HPV, whereas *Mycoplasma* sp. was linked to reduced contraceptive use. In addition, abnormal colposcopic findings were more prevalent in patients positive for *Ureaplasma* spp. and/or *Mycoplasma* sp.

However, more robust studies are needed to explore the interrelationship of *Ureaplasma* and *Mycoplasma* with HPV and preneoplastic lesions.
